# Remote Surgical Discussion of Multivessel Coronary Artery Disease Patients without Surgery on Site-Retrospective Insights

**DOI:** 10.3390/jcm13010103

**Published:** 2023-12-24

**Authors:** Ariel Roguin, Simha-Ron Meisel, Yaniv Levi, Ofer Kobo, Majd Yehia, Naama Amsalem, Rami Abu Fanne

**Affiliations:** Department of Cardiology, Hillel Yaffe Medical Center, Faculty of Medicine, Technion Israel Institute of Technology, Haifa 3200003, Israelmeisel@hy.health.gov.il (S.-R.M.); yanivl@hymc.gov.il (Y.L.); oferk@hymc.gov.il (O.K.); majdyahia88@gmail.com (M.Y.);

**Keywords:** coronary grafts, SYNTAX, concordance, outcomes, heart team

## Abstract

Objective: The heart team approach is highly advocated for in treatment decision making in patients with multivessel disease (MVD). Nevertheless, many centers lack on-site cardiac surgical services (CSS)/formal heart team. Our local alternative is of remote surgical consultation without a structured image sharing platform. In our understanding, the incidence of anatomical complete revascularization (ACR) under this daily practice, and its clinical impact, has not been discussed before. Methods: We analyzed 477 consecutive patients who were surgically revascularized between January 2009 and March 2018 for MVD, after remote surgical consultation. Unstable, late arrival, and ST elevation patients were excluded (*n* = 163). ACR was considered grafting all anatomic lesions > 50%. Syntax score (SS) calculation and ACR categorization were determined by an independent interventionalist using diagnostic angiograms and available operative reports (*n* = 267). Patients’ outcomes were assessed in relation to multiple clinical variables including troponin result and the revascularization status. Results: Three hundred and fourteen patients were included. Mean age was 64 years, and mean SS-II was 27.3 ± 11. At the 4-year follow-up, the observed mortality (11.8% and 12.9%, with troponin-positive and -negative groups, respectively), myocardial infarction (11.8%), and repeat revascularization (9.8%) were higher than those predicted using a nomogram depicting the predicted 4-year mortality as a function of the SYNTAX II Score (5.3%, 8.8%, and 3.5%, respectively, *p* = 0.02). ACR was reported in 33% of 267 available patients’ reports. After multivariate adjustment ACR was the only variable associated with a significant increase in 4-year mortality (12.3% vs. 6.7%, *p* < 0.05). Conclusions: Partial revascularization in the absence of on-site CSS and a structured heart team platform is a frequent occurrence. Not surprisingly, this occurrence was associated with a higher risk for mid-term mortality. An upfront, structured, virtual, heart team interface is mandatory to particularly prioritize the completeness of revascularization when considering the optimal revascularization mode.

## 1. Introduction

Coronary artery disease is a leading cause of morbidity and mortality in Western countries [1]. When significant coronary narrowing involves two or more major coronary arteries it is defined as multivessel disease (MVD). The prevalence of MVD is 45–88% among patients with typical angina, and it has a grim prognosis as these patients are three times more likely to die than patients with single-vessel disease [2,3]. 

Coronary revascularization is indicated in the presence of significant obstructive CAD. The optimal revascularization strategy depends on the complexity of the coronary anatomy, functional tests, the technical feasibility of each approach, comorbidities, socio-economic factors, and on the cultural background and beliefs of the patients. Since both percutaneous and surgical revascularization are highly effective in achieving complete revascularization, there is still an open debate regarding the preferred revascularization management of patients with MVD; although, in cases of diabetes, reduced cardiac function, complex left main disease, and drug nonadherent patients with MVD, surgical revascularization is superior [4]. 

In this complex situation, the international guidelines for coronary revascularization have ascribed the highest level of recommendation (class 1) to the use of a heart team for decision making regarding the optimal treatment of patients with MVD [5,6,7,8,9,10]. In parallel, the 2018 European guidelines on myocardial revascularization [5] have outlined several imperative clues for choosing between CABG and PCI, including (1) calculation of the Society of Thoracic Surgeons (STS) score (class IB) or EuroSCORE II (for in-hospital surgical mortality, class IIb B), (2) the SS-I and SS-II (for anatomical complexity, class IB), and (3) the expected completeness of revascularization using each modality considered (class I B) to minimize residual ischemia [11,12].

The current study is based on consecutive real-world patients treated at a tertiary medical center with no on-site CSS and no formal heart team platform. Our local alternative is remote surgical consultation, lacking an image-sharing platform before patients’ referral to surgery. The achievement of ACR under this policy is ill addressed. In this study, we sought to evaluate the rate of partial revascularization and its long-term consequences. 

## 2. Methods

The current study is a retrospective, observational, single-center cohort-based study that included patients admitted to the Hillel Yaffe Medical Center (HYMC) and referred to bypass surgery for MVD between January 2009 and March 2018. We included only patients who were sent to CABG and underwent CABG; patients who received PCI rather than CABG were not included or specified. All treating interventional cardiologists had more than 10 years of experience. The decision to send for CABG was completely based on anatomical coronary assessment. No physiological coronary tests were applied. HYMC is a university-affiliated hospital located in northern Israel with a catheterization lab operating 24/7 on-call for primary PCI, but it has no on-site CSS. Each patient catheterized undergoes comprehensive assessment including a detailed interview, vital signs measurement, risk factors assessments, blood tests, and echocardiogram at our site. Upon demonstration of MVD anatomy, patients are advised that surgical revascularization should be considered. The procedure is stopped at this stage, and patients are remotely presented by telephone to an off-site cardiac surgeon from one of several receiving centers (according to patient choice and medical insurance coverage). We present basic information including age, vital signs, symptoms, relevant blood tests, neurological status, echocardiographic indices, and the anatomical coronary findings, including our impression considering calcification and the condition of the target vessels for anastomosis. Following presentation, remote surgical approval (or rejection) is obtained, and patients are transferred with no delay/obstacles in this process. In essence, remote patients’ presentation was unstructured and it lacked essential clues for appropriate patients’ assessment including not sharing online diagnostic angiogram (dynamic cine) films, no routine assessment of the in-hospital mortality risk using the STS score/EuroSCORE II, no formal assessment of CAD complexity using the SS-II, and no professional discussion for the feasibility of complete surgical revascularization. ACR was considered as treatment of all coronary artery segments > 1.5 mm in diameter and ≥50% diameter stenosis. The surgeons reviewed the angiographic films at their site; however, no routine preoperative (or postoperative) feedback was given to the referral cardiologist. The study was conducted in accordance with the Declaration of Helsinki and approved by the HYMC Institutional Review Board (protocol code is 79-10-HYMC) with a waiver of the need for informed consent due to its retrospective nature.

**Figure 1:**
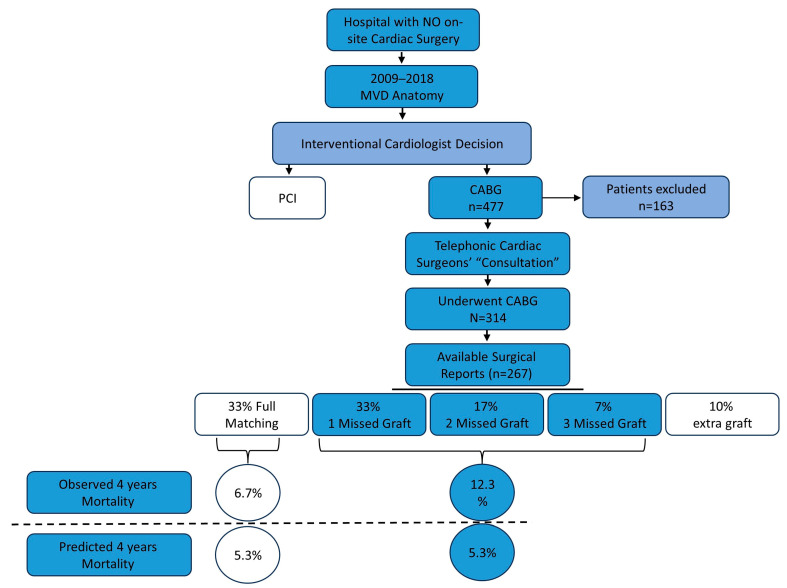
The flowchart of CABG patients’ fate at hospital with no on-site cardiac surgery. MVD—multivessel disease, CABG—coronary artery bypass grafts, and PCI—percutaneous coronary intervention.

All consecutive patients who were referred for CABG during the study period were screened. Patients who experienced out-of-hospital resuscitation, ST elevation myocardial infarction, cardiogenic shock, were hemodynamically unstable, or had significant valvular disease or chronic CHF were excluded. Most coronary bypass surgical reports were obtained from the receiving surgical units (Figure 1, study flowchart). 

### 2.1. Coronary Angiograms and Surgical Reports Evaluation

Coronary anatomy was retrospectively evaluated by two senior interventionists using core laboratory software (QAngio XA; Medis, version 8.0) who provided the SS-I and SS-II. Each patient was assigned a treatment recommendation according to the calculated SS-II: PCI, CABG, and PCI or CABG. Clinical data were obtained from the HYMC medical records. Each patient evaluation included ACR classification using the diagnostic angiograms and provided operative reports. Of note, we had 267 surgical reports available upon request. 

### 2.2. Clinical and Demographic Data

A dataset of potential predictors and confounders including patient demographics, clinical characteristics, and laboratory results were retrieved from the medical records. HYMC operates a fully electronic, medical chart system that includes the clinical, laboratory, and radiological data. It also has access to the Israeli population records; hence, any mortality is automatically updated. 

### 2.3. Study Endpoints

The primary outcome was the frequency of anatomical complete revascularization. The secondary outcomes included the observed 4-year mortality and the incidence of myocardial infarction (MI), repeat revascularization, and stroke among the study population. 

### 2.4. Statistics

All data analyses were performed using IBM SPSS Statistics for Windows, version 25.0 (IBM, Armonk, NY, USA). Baseline demographic, clinical, and angiographic characteristics were summarized using proportions, means (in normally distributed data) or medians (for skewed data), and compared using chi-square statistics for categorical variables and Wilcoxon rank sum tests for continuous variables. Of mention, t-test was used for the mean and Mann–Whitney test for median, when applicable. Multivariant adjustment was completed using the multivariate analysis of variance. A *p*-value of less than 0.05 was considered significant.

## 3. Results

A total of 477 patients referred for CABG between January 2009 and March 2018 were screened. Of these, 163 patients were excluded due to hemodynamic instability, late arrival/ST elevation MI, or significant valvular disease. The remaining 314 patients comprised the study population. Acknowledging plausible negative prognostic value of preoperative cardiac troponin in patients undergoing CABG, patients were divided into troponin-positive (TP, *n* = 144, 45.7%) and troponin-negative (TN, *n* = 170, 54.3%) groups. The TN group included 84 (49.5%) patients with stable angina and 86 (50.5%) patients with unstable angina (baseline patient characteristics shown in Table 1). 

In the TN group, the incidence of previous IHD was significantly higher than in the TP group (81.8% vs. 68.5%, *p* < 0.008) with a relatively lower mean LDL. Table 2 illustrates the coronary anatomical features in both groups, showing that the majority of patients in both groups had three-vessel CAD, with a high incidence of LMCA disease, and chronic total occlusions. 

Next, we compared the SS severity in both groups (Table 3). Both the SS-I and SS-II were non-significantly higher in the TP group (25.4 ± 10.3 vs. 23.6 ± 7.7, *p* = 0.07, and 27.5 ± 10.3 vs. 27.2 ± 11.6, *p* = 0.79, respectively). When stratifying the SS-II recommended modality of revascularization using the troponin status, the recommendations were similar, most being equipoise between CABG and PCI. Using a nomogram depicting predicted 4-year mortality as a function of the SS-II, we predicted a similar 4-year mortality of about 5.3% in both groups. Yet, the 4-year mortality observed in our cohort was 11.8% in the TP group and 12.9% in the TN groups (*p* = NS). The occurrence of myocardial infarction was 11.8% vs. 8.8% (*p* = 0.008), and the rate of recurrent revascularization was 9.8% vs. 3.5% in the TP and TN groups (*p* = 0.022), respectively. The incidence of stroke was similar in both groups. Intuitively, we hypothesized that the high occurrence of equipoise CABG or PCI recommendation relative to CABG recommendation might reflect a low threshold at our site for referring patients to CABG. We, therefore, evaluated patients’ outcomes as a function of the treatment recommendation provided by the SS-II, i.e., CABG vs. equipoise of CABG or PCI. No significant outcome differences were observed related to the recommendation arm provided: the 4-year mortality rate was 13.4% vs. 12.6% (*p* = 0.85), the occurrence of MI was 14.1% vs. 11.3% (*p* = 1.0), the stroke rate was 9% vs. 8.2% (*p* = 1.0), and the rate of recurrent PCI rate was 6% vs. 7.2% (*p* = 0.8) in the recommended CABG vs. equipoise PCI or CABG groups, respectively. 

In an attempt to reconcile the high mortality rate observed relative to the SS-II-predicted mortality, we tested the occurrence of ACR. Upon request, we obtained 267 surgical reports out of 314. The reports disclosed ACR in only 33% of the cases. In detail, one missed graft was found in 33%, two missed grafts in 17%, and three grafts missing in 7% of cases. The missed grafts were related to the RCA (34%), the marginal (30%) and diagonal arteries’ (28%) territories (Table 4). The baseline characteristics of the patients with ACR vs. incomplete revascularization were similar (Table 5), including the calculated SS-II (27.2 ± 6.3 vs. 27.3 ± 8.9, *p* = NS) and the incidence of MI (55% vs. 53%, *p* = NS). Moreover, 9% of the patients had one extra graft implanted deviating from the requirement of ACR. (A total of 44 grafts were labeled as extra, non-recommended grafts: 2.3% to LAD, 29.5% to diagonals, 31.8% to marginals, 22.7% to right, and 13.6% to ramus arteries.) We further tested whether the admission diagnosis, or the echocardiographically estimated ejection fraction were related to the occurrence of incomplete revascularization (Table 6). We found that severe cardiac dysfunction (<30%) was positively associated with graft miss: only 5% of patients with ACR had severe cardiac dysfunction compared to 11% of patients with ≥1 graft missed (*p* < 0.05). The admission diagnosis was not related to incomplete revascularization. 

Careful review of the graft type showed that 41% of the total grafts were LIMA, 19% RIMA, 1% radial, and 40% venous grafts. The use of free RIMA for grafting was observed in 7% of patients, free LIMA in 0.4%, as T graft in 8%, and the application of sequential grafts in 7%. Of note, the LIMA use rate was 97.3%, the average graft number per patient was 2.97, and the use rate of non-arterial grafts was 51.7%.

We then tested the occurrence of long-term outcomes as a function of partial versus complete revascularization. The SS-II predicted 4-year mortality in both groups was around 5.3%. However, the observed mortality was 12.3% in the partial revascularization group compared to 6.7% in patients with ACR at a median follow-up of 7 vs. 18 months, respectively (*p* < 0.05). We found no difference in outcomes between patients with RCA, OM, or RAMUS missed grafts. The occurrence of stroke (15.6% vs. 15.6%, at a median follow-up 32 vs. 23 months, respectively), and repeat revascularization (10.7% vs. 13.3%, at a median follow-up 12 vs. 15 months, respectively) were comparable between groups (*p* = NS). 

## 4. Discussion

Despite major therapeutic and interventional advances, atherosclerotic cardiovascular disease remains a leading cause of mortality and morbidity globally (1). A significant proportion of these patients require surgical/percutaneous revascularization. Since the global population is aging, we are witnessing more patients with complex MVD. In case of a complex MVD, the guidelines advocate for the utilization of the heart team to decide on the optimal treatment strategy [5,13]. The application of a heart team platform based on different professional disciplines has proved its effectiveness in many other circumstances [14,15,16,17,18]. 

Notwithstanding the importance of the heart team approach, the guidelines also stress the critical role of achieving ACR (class IB recommendation), in an attempt to minimize residual ischemia. ACR is of paramount importance as partial anatomic revascularization was linked to adverse cardiovascular outcomes [19]. Notably, the COURAGE trial nuclear sub-study showed a significant decrease in mortality and myocardial infarction by lowering the extent of residual ischemia from >10% to ≤5% [11]. Moreover, a meta-analysis of 89,883 patients investigated in controlled-randomized and observational studies showed a significant long-term decrease in mortality, myocardial infarction, and repeat catheterization in patients who were completely revascularized [12]. Of note, this large meta-analysis represents 35 studies in which the overall incidence of incomplete revascularization in the CABG arm was 25%, as opposed to 67% in our study. 

At present, the majority of centers performing PCI had no on-site CSS and no formal heart team platform [20,21]. The alternative for an in-house heart team is a remote multidisciplinary heart team in which relevant data must be presented in a structured manner incorporating essential parameters for patients’ assessment including the STS score or EuroSCORE II (for estimating in-hospital surgical mortality), SS-II (for anatomical complexity and long-term outcome prediction post revascularization), and discussing the chances of achieving full revascularization in PCI vs. CABG. As part of this collaboration an on-line angiographic film must be transmitted to the surgeons. Considering MVD patients, previous studies pointed out a substantial discordance between virtual-heart-team-based decisions and the treatment recommendations of interventional cardiologists from referring centers without on-site CSS [21,22]. These studies tested the bottom-line recommendation as a binary variables: CABG, PCI, or conservative medical management. In the current study based on real-life data from the HYMC, we focused on the subgroup of MVD who were eventually surgically revascularized. In view of the sub-optimal patients’ presentation to the consultant surgeon, which potentially impedes productive/transparent revascularization discussion, we intended to assess the degree of attained ACR and its possible implication. Intriguingly, 67% of the cases were partially revascularized. When we assessed the outcome of the study population after surgery, the 4-year mortality rate was more than twice higher than that predicted using the SS-II; the SS-II combines anatomic and clinical prognostic variables (Graphical abstract). Virtually, this tool was validated in different registries and randomized trials as a reliable predictor of 4-year mortality [23,24]. The observed mortality was also 2-fold higher than the mortality rate in the Freedom trial which evaluated the outcome of patients post CABG [25]. Surprisingly, we found no difference between the partial and complete revascularization regarding repeat revascularization. This observation might be ascribed to the higher early mortality in the partially revascularized group (12.3% compared to 6.7% in patients with full revascularization at a median follow-up of 7 vs. 18 months, respectively, *p* < 0.05). Although previous studies claimed a negative impact of preoperative troponin on outcomes [26], the reported mortality in the present study was not affected by the preoperative troponin status. It was also not explained by the severity of the SS-II, the revascularization mode recommended based on SS-II, or baseline patients’ characteristics. The only parameter positively associated with increased mortality was incomplete anatomical revascularization, which is much higher than the described 25% rate of partial revascularization in most studies [12]. 

**Figure 2:**
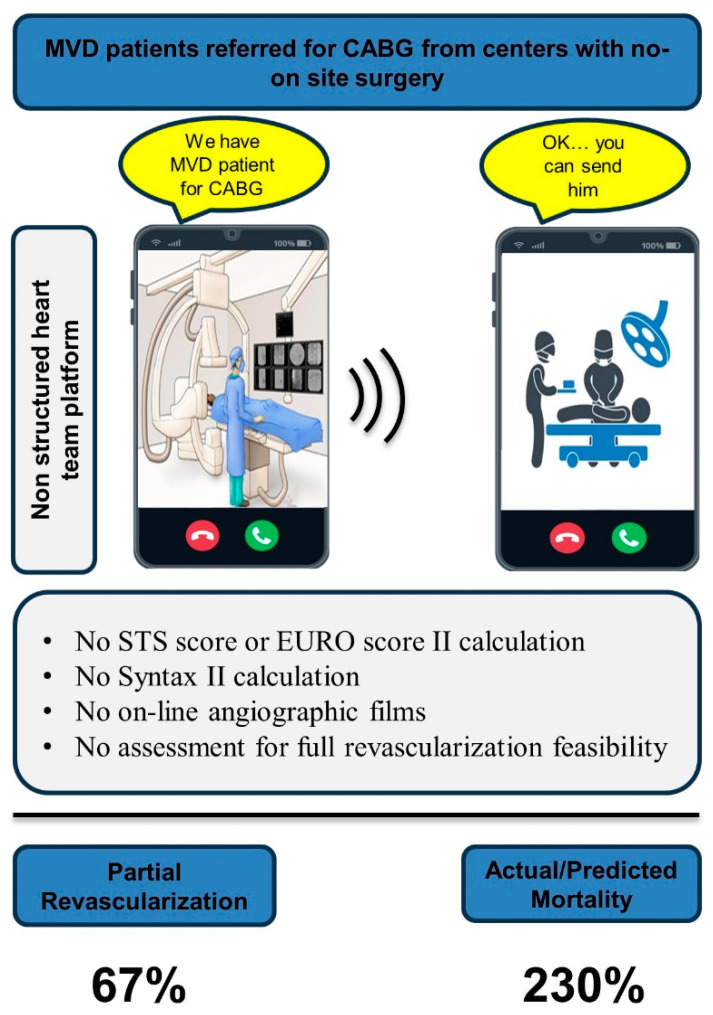
Central picture: local-heart team consequences. MVD—multivessel disease, STS—Society of Thoracic Surgeons.

Possible surgical–technical explanations for the partial revascularization, including the possibility that surgeons view potential targets and the ability to graft a target different than cardiologists, are out of the scope of the present study. A positive association between severe cardiac systolic dysfunction and the incidence of graft miss was observed; nonetheless, the incidence of 4-year mortality among patients with severely reduced cardiac function was the same (40%) with and without graft miss. A previous study showed that the lack of on-site CSS was associated with a 2.5-fold increased likelihood for referral to PCI [21]. Moreover, patients referred to CABG from centers without on-site CSS presented higher SS-II. Nevertheless, our local data, including the mean SS-II exhibited lower threshold for referral to CABG, with a mean SS-II of 24.4 ± 8 vs. 26.7 ± 7 typical of patients surgically revascularized at centers with on-site CSS [21]. A recent study [27] tested the midterm outcomes of MVD patients treated at centers with and without on-site CSS (our site was included in the survey). They found higher 6-year survival probability in centers with on-site CSS (85.1% vs. 81.3%, *p* = 0.04). Intriguingly, although surgical revascularization was associated with a better survival rate relative to percutaneous treatment among patients from hospitals with CSS (89.9% vs. 81.5%, *p* = 0.004), centers with no on-site CSS showed no differences between the two revascularization modes (81.8% vs. 81.1%, *p* = 0.9). Carefully scrutinizing the data shows that there were no survival differences between percutaneously revascularized patients in centers with (81.5%) and without (81.1%) CSS. However, surgically revascularized patients from centers without on-site CSS were negatively impacted (89.9% vs. 81.8%). Acknowledging that both patients’ groups had a similar risk profile, and the operating centers are the same, we surmise that the missing link is related to the heart team implementation. Virtually, non-structured remote patients’ presentation probably missed a critical parameter that must have been addressed prior to patient referral to surgery, which is the expected completion of revascularization by surgery versus PCI. Remote consultant surgeons are not provided with critical clues (including no picture archiving and communications systems to enable high-resolution communication of digital medical images) to anticipate the chances for complete revascularization before patient transfer (see Figure 2 for a graphical abstract of the study). The consultant cardiologist and cardiac surgeon agreed to proceed with surgery; however, the revascularization scheme was not discussed/agreed. Surgeons were provided with the patients’ diagnostic angiographic films at their site before surgery, but they were probably committed to the treatment decision and did not “collegially” activate a local heart team to reconsider treatment options. We believe better MVD patients triage prior to referral would have excluded patients who are more amenable for percutaneous revascularization. In parallel, we believe there are MVD patients treated percutaneously in centers with no on-site CSS that if presented properly by a virtual heart team interface would be found more suitable for surgical revascularization. This reality was superbly reflected in the study by Tsang et al. [24], who tested MVD patients’ triage from a high-volume tertiary care referral center showing that 22.3% of the CABG-recommended patients, and 45.1% the PCI-assigned patients received a different treatment recommendation from the virtual heart teams.

To conclude, the current results demonstrate a high occurrence of incomplete revascularization among MVD patients transferred to cardiothoracic surgery centers. This occurrence was associated with increased mortality. Yet, and despite the former, no causative link can be substantiated based on the current study. Future prospective studies are mandatory to address the above findings. A key message of our study is the urgent need to implement an efficient, state of the art remote heart team approach in centers with no on-site CSS incorporating modalities like PACS (picture archiving and communication system) enabling secure storage and digital transmission of electronic images including the angiographical and echocardiographic films, together with systematic calculation of the SYNTAX and EuroSCORE before patients’ discussion. This approach enhances the ability to assess the feasibility of achieving full revascularization in each approach for better MVD patients’ outcomes. Since the current study, we have ordinarily calculated the SYNTAX and STS/EuroSCOREs for each MVD/complex patient catheterized at our center in the past 4 months, and patients’ angiographical films are simultaneously shared using remote application. Already at the evaluation stage, the scores give an overall impression of the preferred revascularization modality. We believe the current presentation platform is more professional and transparent; however, no conclusions can yet be drawn. 

### Study Limitations

This study has several limitations. First, this was an observational study. Therefore, we cannot generate any causal conclusions. Second, 15% of the surgical reports were missing, which may have introduced bias. However, the baseline characteristics of the remaining patients were similar to those with missed surgical reports, including the occurrence of troponin-positive cases and the mean SS-II. The 4-year observed mortality rate in the missing group was 16% (mean predicted mortality of 5.6% in the whole group, and 6% in the missing group). All the available variables tested (in the missing subgroup) showed no association with increased mortality, including background comorbidities, HbA1c, ejection fraction, and the diagnosis of myocardial infarction. Hence, ascribing the increased mortality to incomplete revascularization seemed reasonable; nevertheless, this assumption is only speculative. Third, there are several reasons that can possibly explain incomplete revascularization including small arteries caliber, diffused atherosclerosis, and distal disease. Yet, possible explanations for incomplete revascularization are out of the scope of our study. Notably, unstable cases were excluded, and thus do not explain grafts’ miss. Lastly, the retrospective design of the study could not address aspects of secondary prevention, achievement of treatment goals for secondary prevention of cardiovascular disease risk factors, and patients’ overall adherence to medications/recommendations. All these parameters were not assessed in our study. Nevertheless, post-surgical treatment plans were uniformly given by the surgical team without referral cardiologists involvements.

## Figures and Tables

**Table 1:** Baseline characteristics of the study patients. TN—troponin negative, DM—diabetes mellitus, HTN—hypertension, HLP—hyperlipidemia, PVD—peripheral vascular disease, IHD—ischemic heart disease, CRP—c-reactive protein, LDL—low density lipoprotein.

	TP (*n* = 144)	TN (*n* = 170)	*p* Value
Age	64.2 ± 9.46	63.9 ± 10.9	*p* = 0.85
Male	119 (82.5%)	133 (80.0%)	*p* = 0.23
DM	79 (54.5%)	91 (53.5%)	*p* = 0.91
HTN	112 (77%)	124 (73%)	*p* = 0.36
HLP	107 (74%)	127 (75%)	*p* = 1.00
Smoke	68 (47%)	88 (52%)	*p* = 0.43
PVD	15 (9.8%)	16 (9.4%)	*p* = 1.00
Renal failure	36 (24.5%)	49 (28.8%)	*p* = 0.44
Chronic IHD	99 (68.5%)	139 (81.8%)	*p* = 0.008
Hemoglobin	13.7 ± 1.8	13.4 ± 1.8	*p* = 0.14
Creatinine	1.06 ± 0.40	1.18 ± 0.78	*p* = 0.094
CRP	5.5 (2.2–14.43)	4.6 (2.4–10.3)	*p* = 0.15
LDL	97.0 (75–130)	86.0 (65.5–122)	*p* = 0.055

**Table 2:** Key coronary anatomical features in the study groups. LMCA—left main coronary artery, LAD—left anterior descending, CTO—chronic total occlusion.

	TP (*n* = 144)	TN (*n* = 170)	*p* Value
No. of diseased vessels			
1	12 (8%)	20 (12%)	
2	43 (30%)	49 (29%)	
3+	90 (62%)	100 (59%)	0.50
LMCA			
Proximal	18 (17%)	18 (15%)	
Distal	34 (33%)	39 (34%)	0.28
Combined	7 (7%)	2 (2%)	
Proximal LAD involvement	82 (57%)	101 (60%)	0.57
No. of lesions per patient	1.53 ± 4.08	1.74 ± 4.13	0.77
CTO	69 (49%)	89 (55%)	0.3

**Table 3:** SYNTAX I and II scores as calculated by two senior interventional cardiologists, including the predicted mortality and recommended treatment modality. PCI—percutaneous coronary intervention, CABG—coronary artery bypass graft.

	TP (*n* = 144)	TN )*n* = 170)	*p* Value
SYNTAX score I	25.4 ± 10.3	23.6 ± 7.7	0.072
SYNTAX score II PCI	33.4 ± 10.9	32.7 ± 11.4	0.59
SYNTAX score II PCI mortality	7.4 (4.2–17.1)	7.7 (4.1–14.3)	0.54
SYNTAX score II CABG	27.5 ± 10.3	27.2 ± 11.6	0.79
SYNTAX score II CABG mortality	5.3 (3.0–9.4)	5.3 (2.9–11.2)	0.89
SYNTAX II Recommendation:			
CABG	54 (37%)	44 (26%)	
PCI	4 (3%)	10 (6%)	
CABG or PCI	86 (60%)	113 (68%)	0.073

**Table 4:** The specification of the missed grafts. LAD—left anterior descending, D—diagonal, OM1—obtuse marginal1, OM2—obtuse marginal2, RCA—right coronary artery, PDA—posterior descending artery, PLA—posterior lateral artery.

Graft Missed	Number	% Total (267)
LAD	5	2%
D	74	28%
OM1	53	20%
OM2	26	10%
RCA	10	4%
PDA	45	17%
PLA	34	13%
RAMUS	19	7%

**Table 5:** Baseline characteristics of the patients with full matching vs. incomplete revascularization. LDL—low density lipoprotein, HDL—high density lipoprotein, CRP—C-reactive protein.

Variable	Full Match	Missed Grafts	*p* Value
Age (years)	66	68	0.9
Males%	71%	81%	0.1
Diabetes	51%	55%	0.08
Renal failure	18%	22%	0.3
Hypertension	76%	66%	0.1
Hyperlipidemia	76%	75%	0.9
Smoking	51%	47%	0.4
LDL	95.6	98.1	0.6
HDL	39.0	38.3	0.4
CRP	14.9	11.5	0.2
Creatinine	1.1	1.1	1
Hemoglobin	13.2	13.6	1

**Table 6:** The cardiac function as estimated using echocardiography in patients with extra graft, missed grafts, and full matching. EF—ejection fraction, TP—troponin positive, TN—troponin negative. *p* value represents the difference between missed and full match groups.

	Extra Graft	Missed Grafts	Full Match	*p* Value
EF < 30%	12%	11%	5%	0.03
EF > 30%	88%	89%	95%	0.04
TP	53%	53%	55%	0.7
TN	47%	48%	45%	0.9

## Data Availability

This study is based on real-world patient data, including demographics and comorbidity factors, that cannot be communicated due to patient privacy concerns.

## Abbreviations and Acronyms

CABG	coronary artery bypass graft
SYNTAX	Synergy Between PCI With Taxus and Cardiac Surgery
MVD	multivessel disease
CAD	coronary artery disease
PCI	percutaneous coronary intervention
MI	myocardial infarction
IHD	ischemic heart disease
LMCA	left main coronary artery
LIMA	left internal mammary artery
RIMA	right internal mammary artery

## References

[1] Alwan A. (2011). Global Status Report on Noncommunicable Diseases 2010.

[2] Chaitman B.R., Bourassa M.G., Davis K., Rogers W.J., Tyras D.H., Berger R., Kennedy J.W., Fisher L., Judkins M.P., Mock M.B. (1981). Angiographic prevalence of high-risk coronary artery disease in patient subsets (CASS). Circulation.

[3] Lopes N.H., Paulitsch F.D.S., Gois A.F., Pereira A.C., Stolf N.A., Dallan L.O., Ramires J.A., Hueb W.A. (2008). Impact of number of vessels disease on outcome of patients with stable coronary artery disease: 5-year follow-up of the Medical, Angioplasty, and bypass Surgery Study (MASS). Eur. J. Cardiothorac. Surg..

[4] Hillis L.D., Smith P.K., Anderson J.L., Bittl J.A., Bridges C.R., Byrne J.G., Cigarroa J.E., DiSesa V.J., Hiratzka L.F., Hutter A.M. (2011). 2011 ACCF/AHA Guideline for Coronary Artery Bypass Graft Surgery: Executive summary: A report of the American College of Cardiology Foundation/American Heart Association Task Force on Practice Guidelines. Circulation.

[5] Neumann F.-J., Sousa-Uva M., Ahlsson A., Alfonso F., Banning A.P., Benedetto U., Byrne R.A., Collet J.-P., Falk V., Head S.J. (2019). ESC Scientific Document Group. 2018 ESC/EACTS guidelines on myocardial revascularization. Eur. Heart J..

[6] Teo K.K., Cohen E., Buller C., Hassan A., Carere R., Cox J.L., Ly H., Fedak P.W., Chan K., Légaré J.F. (2014). Canadian Cardiovascular Society/Canadian Association of Interventional Cardiology/Canadian Society of Cardiac Surgery position statement on revascularization—Multivessel coronary artery disease. Can. J. Cardiol..

[7] Fihn S.D., Blankenship J.C., Alexander K.P., Bittl J.A., Byrne J.G., Fletcher B.J., Fonarow G.C., Lange R.A., Levine G.N., Maddox T.M. (2014). 2014 ACC/AHA/AATS/PCNA/SCAI/STS focused update of the guideline for the diagnosis and management of patients with stable ischemic heart disease: A report of the American College of Cardiology/American Heart Association Task Force on Practice Guidelines, and the American Association for Thoracic Surgery, Preventive Cardiovascular Nurses Association, Society for Cardiovascular Angiography and Interventions, and Society of Thoracic Surgeons. J. Am. Coll. Cardiol..

[8] Long J., Luckraz H., Thekkudan J., Maher A., Norell M. (2012). Heart team discussion in managing patients with coronary artery disease: Outcome and reproducibility. Interact. Cardiovasc. Thorac. Surg..

[9] Yamasaki M., Abe K., Horikoshi R., Hoshino E., Yanagisawa H., Yoshino K., Misumi H., Mizuno A., Komiyama N. (2019). Enhanced outcomes for coronary artery disease obtained by a multidisciplinary heart team approach. Gen. Thorac. Cardiovasc. Surg..

[10] Patterson T., McConkey H.Z., Ahmed-Jushuf F., Moschonas K., Nguyen H., Karamasis G.V., Perera D., Clapp B.R., Roxburgh J., Blauth C. (2019). Long-term outcomes following heart team revascularization recommendations in complex coronary artery disease. J. Am. Heart Assoc..

[11] Shaw L.J., Berman D.S., Maron D.J., Mancini G.J., Hayes S.W., Hartigan P.M., Weintraub W.S., O’Rourke R.A., Dada M., Spertus J.A. (2008). Optimal medical therapy with or without percutaneous coronary intervention to reduce ischemic burden: Results from the Clinical Outcomes Utilizing Revascularization and Aggressive Drug Evaluation (COURAGE) trial nuclear substudy. Circulation.

[12] Garcia S., Sandoval Y., Roukoz H., Adabag S., Canoniero M., Yannopoulos D., Brilakis E.S. (2013). Outcomes after complete versus incomplete revascularization of patients with multivessel coronary artery disease: A meta-analysis of 89,883 patients enrolled in randomized clinical trials and observational studies. J. Am. Coll. Cardiol..

[13] Lawton J.S., Tamis-Holland J.E., Bangalore S., Bates E.R., Beckie T.M., Bischoff J.M., Bittl J.A., Cohen M.G., DiMaio J.M., Don C.W. (2022). 2021 ACC/AHA/SCAI Guideline for Coronary Artery Revascularization: Executive Summary: A Report of the American College of cardiology/American Heart association Joint Ie on Clinical Practice Guidelines. Circulation.

[14] Woolley A.W., Chabris C.F., Pentland A., Hashmi N., Malone T.W. (2010). Evidence for a collective intelligence factor in the performance of human groups. Science.

[15] Wolf M., Krause J., Carney P.A., Bogart A., Kurvers R.H. (2015). Collective intelligence meets medical decision-making: The collective outperforms the best radiologist. PLoS ONE.

[16] Cooper D.J., Kagel J.H. (2005). Are two heads better than one? team versus individual play in signaling games. Am. Econ. Rev..

[17] Koriat A. (2012). When are two heads better than one and why?. Science.

[18] Kurvers R.H., Herzog S.M., Hertwig R., Krause J., Carney P.A., Bogart A., Argenziano G., Zalaudek I., Wolf M. (2016). Boosting medical diagnostics by pooling independent judgments. Proc. Natl. Acad. Sci. USA.

[19] Farooq V., Serruys P.W., Garcia-Garcia H.M., Zhang Y., Bourantas C.V., Holmes D.R., Mack M., Feldman T., Morice M.C., Ståhle E. (2013). The negative impact of incomplete angiographic revascularization on clinical outcomes and its association with total occlusions: The SYNTAX (Synergy Between Percutaneous Coronary Intervention with Taxus and Cardiac Surgery) trial. J. Am. Coll. Cardiol..

[20] Rashid M., Ludman P.F., Mamas M.A. (2019). British cardiovascular intervention society registry framework: A quality improvement initiative on behalf of the National Institute of Cardiovascular Outcomes Research (NICOR). Eur. Heart J. Qual. Care Clin. Outcomes.

[21] Ram E., Goldenberg I., Kassif Y., Segev A., Lavee J., Shlomo N., Raanani E. (2018). Comparison of patients with multivessel disease treated at centers with and without on-site cardiac surgery. J. Thorac. Cardiovasc. Surg..

[22] Tsang M.B., Schwalm J.D., Gandhi S., Sibbald M.G., Gafni A., Mercuri M., Salehian O., Lamy A., Pericak D., Jolly S. (2020). Comparison of Heart Team vs. Interventional Cardiologist Recommendations for the Treatment of Patients with Multivessel Coronary Artery Disease. JAMA Netw. Open.

[23] Farooq V., Van Klaveren D., Steyerberg E.W., Meliga E., Vergouwe Y., Chieffo A., Kappetein A.P., Colombo A., Holmes D.R., Mack M. (2013). Anatomical and clinical characteristics to guide decision making between coronary artery bypass surgery and percutaneous coronary intervention for individual patients: Development and validation of SYNTAX score II. Lancet.

[24] Campos C.M., van Klaveren D., Iqbal J., Onuma Y., Zhang Y.J., Garcia-Garcia H.M., Morel M.A., Farooq V., Shiomi H., Furukawa Y. (2014). Predictive Performance of SYNTAX Score II in Patients With Left Main and Multivessel Coronary Artery Disease-analysis of CREDO-Kyoto registry. Circ. J..

[25] Bansilal S., Farkouh M.E., Hueb W., Ogdie M., Dangas G., Lansky A.J., Cohen D.J., Magnuson E.A., Ramanathan K., Tanguay J.-F. (2012). The future REvascularization Evaluation in patients with Diabetes mellitus: Optimal management of Multivessel disease (FREEDOM) trial: Clinical and angiographic profile at study entry. Am. Heart J..

[26] Thielmann M., Massoudy P., Neuhauser M., Tsagakis K., Marggraf G., Kamler M., Mann K., Erbel R., Jakob H. (2006). Prognostic value of preoperative cardiac troponin I in patients undergoing emergency coronary artery bypass surgery with non-ST-elevation or ST-elevation acute coronary syndromes. Circulation.

[27] Ram E., Raanani E., Klempfner R., Peled Y., Sternik L., Segev A. (2022). Midterm outcomes of patients with multivessel disease treated at centers with and without on-site cardiac surgery services. J. Thorac. Cardiovasc. Surg..

